# Identification of an iridium(III) complex with anti-bacterial and anti-cancer activity

**DOI:** 10.1038/srep14544

**Published:** 2015-09-29

**Authors:** Lihua Lu, Li-Juan Liu, Wei-chieh Chao, Hai-Jing Zhong, Modi Wang, Xiu-Ping Chen, Jin-Jian Lu, Ruei-nian Li, Dik-Lung Ma, Chung-Hang Leung

**Affiliations:** 1 Department of Chemistry, Hong Kong Baptist University, Kowloon Tong, Hong Kong, China; 2 State Key Laboratory of Quality Research in Chinese Medicine, Institute of Chinese Medical Sciences, University of Macau, Macao, China; 3 Department of Biomedical Science and Environmental Biology, College of Life Science, Kaohsiung Medical University, Kaohsiung, Taiwan

## Abstract

Group 9 transition metal complexes have been widely explored as therapeutic agents due to their unique geometry, their propensity to undergo ligand exchanges with biomolecules and their diverse steric and electronic properties. These metal complexes can offer distinct modes of action in living organisms compared to carbon-based molecules. In this study, we investigated the antimicrobial and anti-proliferative abilities of a series of cyclometallated iridium(III) complexes. The iridium(III) complex **1** inhibited the growth of *S. aureus* with MIC and MBC values of 3.60 and 7.19 μM, respectively, indicating its potent bactericidal activity. Moreover, complex **1** also exhibited cytotoxicity against a number of cancer cell lines, with particular potency against ovarian, cervical and melanoma cells. This cyclometallated iridium(III) complex is the first example of a substitutionally-inert, Group 9 organometallic compound utilized as a direct and selective inhibitor of *S. aureus*.

The design and synthesis of metal complexes for imaging and therapy have attracted considerable attention from inorganic medicinal chemists over the past decade^1^,^2^,^3^,^4^. Increasingly, transition metal complexes have been explored as therapeutic agents for treating human diseases due their prominent advantages. For example, the steric and electronic properties of metal complexes can be fine-tuned by modification of their auxiliary ligands. Moreover, metal complexes possess unique geometries for the attachment of ligands, and they can also undergo ligand exchange reactions with specific biomolecules^5^. Consequently, metal complexes can offer distinct modes of action compared to carbon-based molecules^6^,^7^,^8^,^9^,^10^,^11^,^12^,^13^,^14^,^15^,^16^.

With the global rise of antibiotic resistance and the lack of new antibiotics reaching the market, new antimicrobial drugs with novel mechanisms of action are desperately needed^17^. Complexes of copper(II)^18^, silver(I)^19^,^20^,^21^, ruthenium(II)^22^, bismuth(III)^23^ vanadium(IV)^24^, and iron(II)^25^, among other complexes^26^,^27^,^28^,^29^,^30^,^31^,^32^,^33^,^34^,^35^,^36^, have been reported to disrupt antibiotic-resistant biofilms, exert synergistic bactericidal activity with other biocides, inhibit metabolic pathways in a selective manner or to kill multidrug-resistant bacteria.

A number of mechanisms of metal-induced microbial activity have been extensively studied^37^. The first is the production of reactive oxygen species (ROS) and antioxidant depletion. Treatment of *E. coli* with exogenous hydrogen peroxide (H_2_O_2_) or agents that catalyse the generation of superoxide (O_2_^•−^) resulted in DNA damage and the inhibition of enzyme activities that are important for bacterial growth^37^. Moreover, bacterial and *S. cerevisiae* strains lacking ROS-scavenging enzymes or other cellular antioxidants showed altered sensitivity towards redox-active metals^38^. Alternatively, certain metals might disrupt cellular donor ligands that coordinate Fe, leading to the release of additional Fenton-active Fe into the bacterial cytoplasm resulting in increased ROS production. Oxidative stress in bacteria can also lead to the oxidation of cellular thiols, leading to the formation of protein disulphides and the depletion of antioxidant reserves such as glutathione in bacterial cells^39^.

Another interesting strategy is the use of “Trojan horse” compounds that trick bacteria into taking up toxic metals. These ions can be delivered to bacterial cells by conjugation to a siderophore such as desferrioxamine (DFO)^40^, enterochelin^41^ or protoporphyrin IX (PPIX)^42^. Notably, gallium nitrate (Ga(NO_3_)_3_), Ga-DFO and Ga-PPIX show broad-spectrum bactericidal activity against Gram-negative and Gram-positive bacteria *in vitro* and *in vivo* of infection^43^,^44^.

Only limited examples of kinetically-inert Group 9 metal complexes have been reported as antibacterial and anticancer agents^45^,^46^,^47^. Recently, three cyclometalated iridium(III) complexes bearing dithiocarbamate derivatives have been reported to interact with calf thymus DNA and possess anti-bacterial activity^48^. The delocalization of the π-electrons over the chelate ring was proposed to increase the lipophilicity of the complexes and facilitate their penetration into bacterial cell membranes, thereby resulting in inhibition of bacterial growth. Highly hydrophobic Ir(III) complexes containing both Cp* and a C,N-chelating ligand have been validated their significant cytotoxic activity towards A2780 human ovarian cancer cells^49^. Additionally, an NHC iridium(I) complex with significant antiproliferative properties *in vitro* has been shown to target cytochrome c with simultaneous oxidation of Ir(I) to Ir(III)^50^. In this report, we describe the synthesis and biological evaluation of a substitutionally-inert, organometallic iridium(III) complex **1** (Fig. 1) that inhibits the growth of *Staphylococcus aureus*.

## Results and Discussion

### Synthesis

The iridium(III) complexes **1–4** and rhodium(III) complex **5** are kinetically-inert, organometallic complexes with a general structure [M(C^N)_2_(N^N)]^+^ (M = Ir or Rh). Complexes **1** and **2** contain a 5-amino-1, 10-phenathroline N^N ligand, while complexes **3–5** contain the bathophenanthroline N^N ligand. In regards to the C^N ligand, complexes **3** and **5** possess 2-phenylpyridine ligands, while complex **1** bears the related 2-(*p*-tolyl)pyridine ligand. Complexes **2** and **4** carry the bulkier 2-phenylquinoline and 1-isoquinoline C^N ligands, respectively.

The syntheses and characterization of the iridium(III) or rhodium(III) complexes **1**–**5** are described in the Methods section. The spectroscopic data of the complexes are presented in Table 1. Complexes **2** and **5** are novel whereas complexes **1** 51, **3** 52 and **4** 53 have been previously described. All complexes were synthesized and tested as racemic mixtures of enantiomers.

### Antibacterial activity

**Figure 2:**
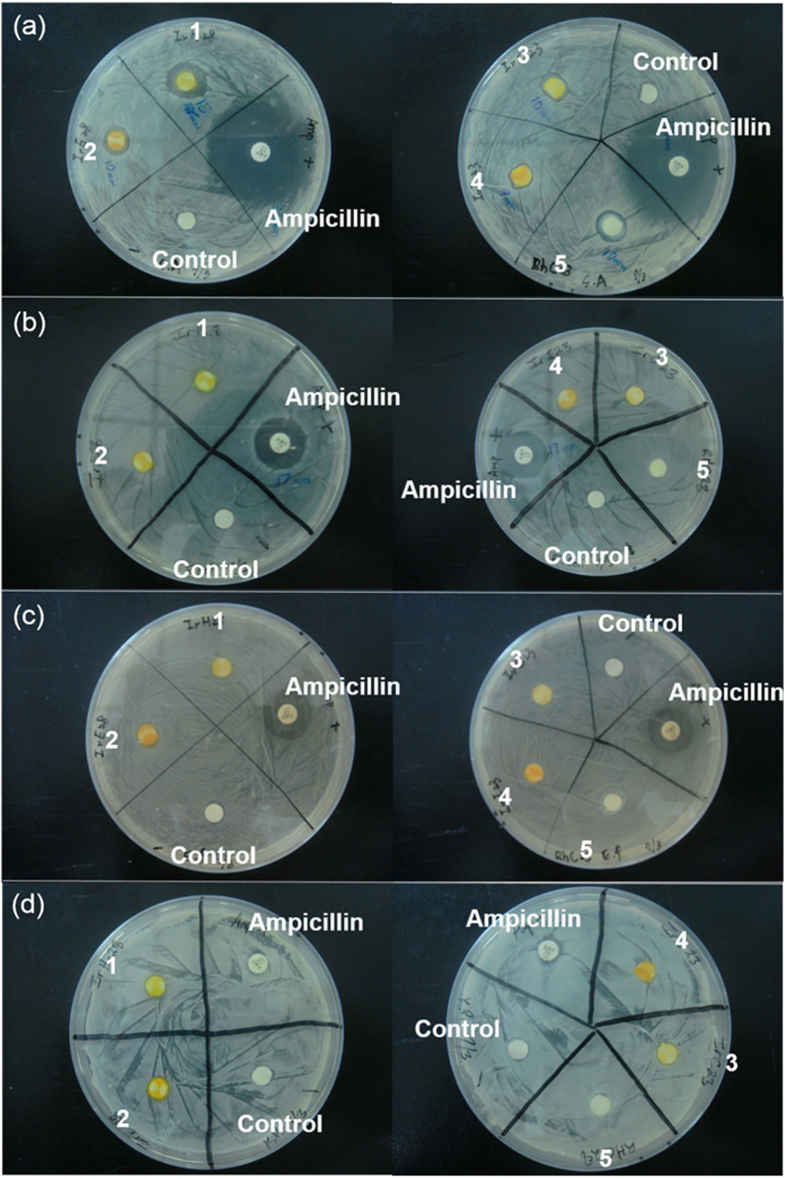
Anti-bacterial activity of complexes 1–5 as determined by the disk diffusion assay. (**a**) The anti-*S. aureus* activity of complexes **1**–**5**. (**b**) The anti-*E. coli* activity of complexes **1**–**5**. (**c**) The anti-*E. faecalis* activity of complexes **1**–**5**. (**d**) The anti-*K. pneumoniae* activity of complexes **1**–**5**. All complexes used are racemic mixture of enantiomers.

The anti-bacterial activity of the complexes **1**–**5** was investigated using a disk diffusion assay. In this assay, four different bacterial strains (*Staphylococcus aureus*, *Escherichia coli*, *Enterococcus faecalis* and *Klebsiella pneumoniae*) were plated on an agar dish, and disks soaked with solutions of the metal complexes (11.5 μM) or ampicillin were placed onto the dish. The antimicrobial activity of the metal complexes would be manifested as the absence of bacterial growth around the disk, due to diffusion of the compounds into the surrounding agar. After 24 h, the growth inhibition zones of the test strains were determined. Encouragingly, the results showed that racemic complex **1** (*rac*-**1**) exhibited selective anti-bacterial activity towards *S. aureus*, with an average inhibition zone of 15 mm (Fig. 2). On the other hand, the other metal complexes showed limited antibacterial activity.

### Determination of MIC and MBC values

**Figure 3:**
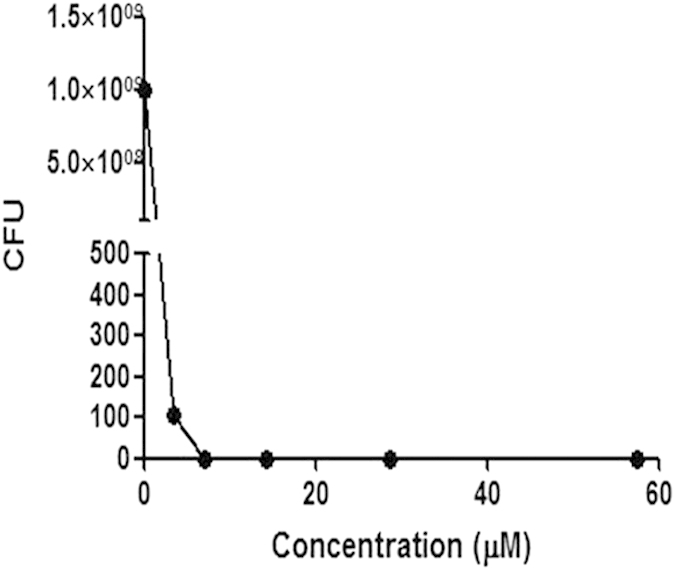
Inhibition of the growth of *S. aureus* by racemic mixture of complex **1** as determined by the broth dilution method. Results are representative of three independent experiments, and the complete data are shown in Table S2.

We next determined the minimum inhibitory concentration (MIC) value of complex **1** against *S. aureus* by the broth dilution method. Briefly, *S. aureus* inoculum in Luria Bertain broth was incubated with serial dilutions of complex **1** at 37 °C for 24  h. The MIC was determined as the lowest concentration of complex **1** inhibiting visible bacterial growth. The results showed that the MIC of *S. aureus* grown with complex **1** was 3.60 μM (Fig. 3).

To determine the MBC (minimum bactericidal concentration), the agar dilution method was used. Briefly, *S. aureus* inoculum treated with different concentrations of complex **1** were taken aseptically from tubes that had not presented visible turbidity and plated onto agar. The MBC was considered as the lowest concentration of complex **1** that allowed less than 0.1% of the original inoculum treated with complex **1** to grow on the surface of the agar. The results showed that the MBC of *S. aureus* grown with complex **1** was 7.19 μM. To determine the nature of antibacterial effect of a compound, the MBC/MIC ratio is typically used^54^. When this ratio is lower than 4, the compound is considered as a bactericidal. On the other hand, if this ratio is higher than 4, the compound is considered to be bacteriostatic. As the MBC/MIC ratio of complex **1** is 2, complex **1** is considered to be bactericidal.

From these data, a brief structure-activity relationship analysis can be conducted. Complex **1** contains the 2-(*p*-tolyl)pyridine ligand C^N, which is relatively smaller compared to the quinoline or isoquinoline-based C^N ligands of complexes **2** and **4**, respectively. This suggests that smaller C^N ligands may be beneficial for antimicrobial activity. Additionally, complex **1** also contains an amino substituent on the phenanthroline N^N ligand. The amino group may confer the necessary hydrophilic/hydrophobic balance for the complex to penetrate the thick peptidoglycan cell wall of Gram-positive bacteria such as *S. aureus*. While complex **2** contains the same 5-amino-1,10-phenanthroline N^N substituent as complex **1**, its larger 2-phenylquinoline C^N ligand may have diminished its activity.

Interestingly, a slightly increased zone area was observed when the iridium(III) center of **3** was replaced with rhodium(III) (as in congener **5**), suggesting the importance of the metal center in contributing to antibacterial activity. However, complexes **3**–**5**, containing the relatively large bathophenanthroline N^N ligand, generally showed modest zone areas and were less active compared to complex **1**. In particular, complexes **2**–**5** showed only very limited activity against *S. aureus*. Additionally, complexes **2**–**5** at a concentration of 50–60 μM were not able to inhibit bacterial growth in LB broth. These complexes might exhibit antibacterial activity at higher concentrations, but this would not be practical for clinical use. Taken together, these findings suggest that the presence of the relatively bulky N^N ligand detracts from antibacterial activity. We propose that the lipophilicity and/or structure of complex **1** might allow it to effectively pass through the hydrophobic interior of the bacterial cell membrane.

### Partition Coefficients (log *P*)

The octanol-water partition coefficients (log *P*) for complex **1** was determined. Complex **1** showed a log *P* value of 0.629, indicating that it is slightly lipophilic and also satisfies Lipinski’s lipophilicity criterion (log *P* < 5) for drug likeness.

### Evaluation of cell proliferation

**Figure 4:**
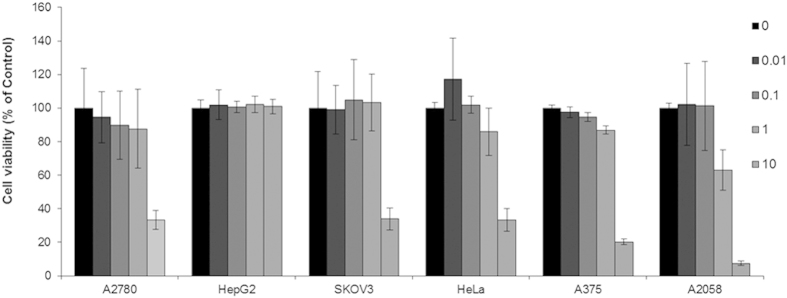
Cell viability of racemic mixture of complex **1** against A2780, HepG2, SKOV3, HeLa, A375 and A2058 cells as determined by the MTT assay. Error bars represent the standard deviations of the results from three independent experiments.

Lipophilicity correlates with cytotoxic potency. Therefore, the cytotoxicity of complex **1** towards different cell lines was evaluated. By using the 3-(4,5-dimethylthiazol-2-yl)-2,5-diphenyltetrazolium bromide (MTT) assay, the *in vitro* cytotoxicity of complex **1** was evaluated in a panel of human cell lines, including ovarian cancer A2780 and SKOV3 cells, hepatocellular carcinoma HepG2 cells, cervical adenocarcinoma HeLa cells, and malignant melanoma A375 and A2058 cells. Cells were exposed to complex **1** in concentrations ranging from 0.01 to 10 μM and cellular proliferation was assessed after 72 h. The results showed that complex **1** inhibited cellular proliferation of A2780, HepG2, SKOV3, HeLa, A375, and A2058 cells in a dose-dependent manner with IC_50_ values of 7.23 ± 1.30, 17.0 ± 1.39, 1.77 ± 1.70, 1.68 ± 1.29, 1.55 ± 0.15 and 1.24 ± 0.31 μM, respectively (Fig. 4). The results indicate that the cytotoxic effect of complex **1** is cell type-dependent. Complex **1** selectively inhibits growth of ovarian, cervical and melanoma cells, but not hepatocarcinoma cells.

### Stability of complex 1

**Figure 5:**
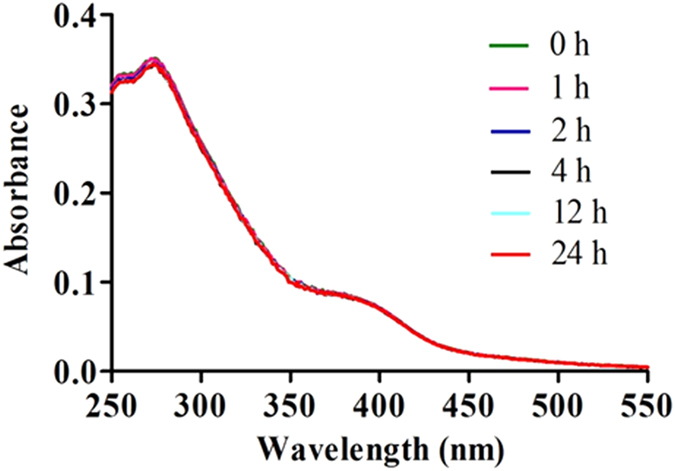
UV/Vis absorption of 1 (2.5 μM) in H_2_O/acetonitrile (400:1) at t* *= 0 h and after incubation for 0, 1, 2, 4, 12, and 24 h at 298 K.

The most potent complex **1** was stable in H_2_O/acetonitrile (400:1) solution at 298 K for at least 24 h as revealed by UV-Vis spectroscopy. No significant change in absorption spectrum of complex **1** was observed over 24 h (Fig. 5). The result indicates that complex **1** is stable at room temperature.

## Methods

### Materials and cell lines

All chemicals, unless specified, were obtained from Sigma-Aldrich. *Staphylococcus aureus*, *Enterococcus faecalis*, *Escherichia coli*, *Klebsiella pneumonia*, HepG2, SKOV3, HeLa, A375 and A2058 were purchased from American Type Culture Collection (ATCC). A2780, HepG2, SKOV3, HeLa, A375 and A2058 cells were routinely grown in suspension in 90% RPMI-1640 containing L-glutamine (2 nM) or DMEM, antibiotics (100 IU penicillin/mL, 100 μg streptomycin/mL) and supplemented with 10% (v/v) fetal bovine serum (FBS). The cells were maintained at a cell density of 0.2–1 × 10^6^ cells/mL in a 5% CO_2_ humidified atmosphere at 37 °C.

### General experimental

Mass spectrometry was performed at the Mass Spectroscopy Unit at the Department of Chemistry, Hong Kong Baptist University, Hong Kong (China). Melting points were determined using a Gallenkamp melting apparatus and are uncorrected. Deuterated solvents for NMR purposes were obtained from Armar and used as received.

^1^H and ^13^C NMR were recorded on a Bruker Avance 400 spectrometer operating at 400 MHz (^1^H) and 100 MHz (^13^C). ^1^H and ^13^C chemical shifts were referenced internally to solvent shift (acetone-*d*_6_: ^1^H δ 2.05, ^13^C δ 29.8, 206.1). Chemical shifts (δ) are quoted in ppm, the downfield direction being defined as positive. Uncertainties in chemical shifts are typically ±0.01 ppm for ^1^H and ±0.05 for ^13^C. Coupling constants are typically ±0.1 Hz for ^1^H-^1^H and ±0.5 Hz for ^1^H-^13^C couplings. The following abbreviations are used for convenience in reporting the multiplicity of NMR resonances: s, singlet; d, doublet; t, triplet; m, multiplet. All NMR data was acquired and processed using standard Bruker software (Topspin).

### Photophysical measurement

Emission spectra and lifetime measurements for complex **1** were performed according to the previously described method^53^.

### Synthesis

These complexes were synthesized using a modified literature method^55^. MCl_3·_3H_2_O was heated to 150 °C with 2.2 equivalents of C^N ligand in 3:1 methoxymethanol and deionized water under a nitrogen atmosphere for 12 h. The product was filtered off and washed with ether (3 × 50 mL) and then with deionized water (3 × 50 mL). A suspension of [M_2_(C^N)_4_Cl_2_] (0.2 mmol) and the N^N ligand (0.44 mmol) in a mixture of dichloromethane:methanol (1:1, 20 mL) was refluxed overnight under a nitrogen atmosphere. The resulting solution was then allowed to cool to room temperature, and filtered to remove unreacted cyclometallated dimer. To the filtrate, a solution of ammonium hexafluorophosphate (0.5 g in 5 mL methanol) was added and the filtrate was reduced in volume by rotary evaporation until precipitation of the crude product occurred. The precipitate was then filtered and washed with several portions of water (2 × 50 mL) followed by diethyl ether (2 × 50 mL). The product was recrystallized by acetonitrile:diethyl ether vapor diffusion to yield the titled compound. All complexes were characterized by ^1^H-NMR, ^13^C-NMR, high resolution mass spectrometry (HRMS) and elemental analysis.

#### Complex 1

Reported^51^.

#### Complex 2

Yield: 61%. ^1^H NMR (400 MHz, Acetone-*d*_6_) δ 9.22 (d, *J *= 8.4 Hz, 1H), 9.12 (s, 1H), 9.06 (d, *J *= 8.0 Hz, 1H), 8.98 (d, *J *= 4.0 Hz, 1H), 8.94 (d, *J *= 4.0 Hz, 1H), 8.59 (d, *J *= 8.8 Hz, 2H), 8.52 (d, *J *= 8.8 Hz, 2H), 8.34 (d, *J *= 8.0 Hz, 2H), 8.31-8.25 (m, 2H), 7.84 (d, *J *= 8.0 Hz, 2H), 7.34-7.25 (m, 6H), 6.93 (dd, *J *= 6.0 Hz, 1.2 Hz, 2H), 6.70 (d, *J *= 8.0 Hz, 2H); ^13^C NMR (100 MHz, Acetone-*d*_6_) δ 171.4, 152.6, 152.1, 149.4, 148.6, 148.4, 147.2, 147.1, 145.3, 144.5, 141.9, 141.1, 141.0, 136.1, 135.6, 135.5, 134.1, 133.8, 131.5, 131.4, 131.3, 130.1, 130.0, 128.6, 128.3, 128.2, 127.6, 127.5, 127.3, 126.4, 125.3, 125.2, 124.7, 123.8, 123.7, 119.0, 118.9, 103.7. HRMS: Calcd. for C_42_H_29_IrN_5_[M–PF_6_]^+^: 796.2052 Found: 796.2091. Anal. (C_42_H_29_N_5_IrPF_6_+2H_2_O): C, H, N: calcd. 51.64, 3.40, 7.17, found: 51.65, 3.15, 7.41. The elemental analysis result (Figure S1) was shown in the supporting information.

#### Complex 3

Reported^52^.

#### Complex 4

Reported^53^.

#### Complex 5

Yield: 58%. ^1^H NMR (400 MHz, Acetone-*d*_6_) δ 8.58 (d, *J *= 5.2 Hz, 2H), 8.33-8.30 (m, 4H), 8.06-8.02 (m, 6H), 7.80 (d, *J *= 4.8 Hz, 2H), 7.70-7.66 (m, 10H), 7.19 (t, *J *= 3.6 Hz, 2H), 7.09 (dd, *J *= 6.0 Hz, 1.2 Hz, 4H), 6.52 (d, *J *= 7.6 Hz, 2H); ^13^C NMR (100 MHz, Acetone-*d*_6_) δ 164.6, 164.3, 153.7, 150.0, 148.9, 145.8, 139.3, 137.4, 135.5, 133.4, 129.8, 129.6, 129.4, 129.0, 128.1, 126.9, 126.8, 125.7, 124.1, 123.1, 121.4; HRMS: Calcd. for C_46_H_32_RhN_4_[M–PF_6_]^+^: 743.1682 Found: 743.2193. Anal. (C_46_H_32_N_4_RhPF_6_+0.5H_2_O): C, H, N: calcd. 61.55, 3.71, 6.24, found: 61.51, 3.66, 6.28. The elemental analysis result (Figure S2) was shown in the supporting information.

### Disk diffusion susceptibility test

The disk diffusion method was used to determine sensitivity of bacteria to complexes **1**–**5**. *S. aureus*, *E. faecalis*, *E. coli* and *K. pneumoniae* direct colony suspensions were prepared in Luria Bertain broth for less than 18 h. The turbidity of four bacterial suspensions were standardized to match 1.0 × 10^8^  UFC/mL (or 0.5 McFarland turbidity units). A sterile swab dipped in fresh bacterial suspensions was inoculated to the entire surface of Mueller-Hinton agar and the inoculum was distributed evenly. The plates were incubated with standardizing inoculum suspension for 15 min at room temperature. 10 μL complexes **1**–**5** (11.5 μM) or ampicillin (28 μM) were impregnated on sterile 6 mm paper disks for 15 min. Subsequently, the paper disks were aseptically placed on and pressed down firmly to the inoculated plates. Plates were incubated for 24 h in a 37 °C incubator. After the incubation, the inhibition zones were measured. Ampicillin was used as a positive control for bacterial inhibition. The presence or absence of growth around the disks was a measure of the ability of complexes **1**–**5** to inhibit growth of bacterial. All experiments were done in triplicate.

#### MIC and MBC testing

Complex **1** was examined for its antimicrobial activity against *S. aureus*. The MIC was estimated by the broth dilution method in Luria Bertain broth using the standardized method^56^,^57^,^58^,^59^,^60^. Briefly, serial dilutions of complex **1** were prepared in distilled water with concentrations ranging from 1.8 to 57.6 μM. 1 mL of an *S. aureus* inoculum (1 × 10^8 ^UFC/mL) and 0.1 mL of serially diluted complex **1** were added to 2.9 mL of Luria Bertain broth. Controls without complex **1** were also prepared. After 24 h of incubation at 37 °C under agitation in hermetic tubes, the MIC was determined as the lowest concentration of complex **1** that inhibited visible bacterial growth. The experiments were performed in triplicate. The complete data are shown in Table S2 in the Supplementary Information.

To determine the MBC, 10 μL of bacterial inoculum treated with different concentrations of complex **1** was taken aseptically from tubes that had not presented visible turbidity and was plated onto Mueller-Hinton agar. The MBC was considered as the lowest concentration of complex **1** that allowed less than 0.1% of the original inoculum treated with complex **1** to grow on the surface of the Mueller-Hinton agar. The experiments were performed in triplicate. The complete data are shown in Table S2 in the Supplementary Information.

To determine the nature of antibacterial effect of complex **1**, the MBC/MIC ratio was used; when the ratio was lower than 4, complex **1** was considered as a bactericidal complex **1** and when the ratio was higher than 4, it was considered as a bacteriostatic complex **1**.

### Log *P* Determination

This experiment was carried out according to a reported method and the partitions were detected by HPLC^61^. The partition coefficients of the Ir(III) complexes were calculated using the equation log *P* = log([Ir]_WSO_/[Ir]_OSW_), where [Ir]_WSO_ means the concentration of Ir(III) complex dissolved in octanol-saturated water (WSO), and [Ir]OSW means the concentration of Ir(III) complex dissolved in water-saturated octanol (OSW).

### Cell cytotoxicity

Complex **1** was dissolved in dimethylsulfoxide (DMSO) at a stock concentration of 10 mM and serially diluted with low FBS medium to the working concentrations. The evaluation of cells proliferation was conducted using MTT assay. In brief, the cells were seeded in 96-well plates at a density of 8 × 10^4^ cells/mL in complete medium for overnight. Serial dilution of complex **1** in low FBS medium was then added to the wells. After 72 h, 20 μL MTT (5 mg/mL) was added to each well. The plates were then incubated for an additional 4 h at 37 °C while avoiding direct light. Subsequently, the medium in each well was removed and 100 μL DMSO was added to each well. The plates were shaken gently for 10 min at room temperature to completely dissolve the purple formazan in the mitochondria of living cells. The color intensity was measured at 570 nm using a SpectraMax M5 microplate reader (Molecular Devices).

## Conclusion

In summary, we have identified a metal-based complex that selectively inhibited the growth of *S. aureus*. The iridium(III) or rhodium(III) complexes **1**–**5** were synthesized by the reaction of MCl_3·_*n*H_2_O with the C^N ligand to yield the dimeric compound [M_2_(C^N)_4_Cl_2_], which was treated with the corresponding N^N ligand to give the targeted complexes. Complexes **2** and **5** are novel and have been characterized for the first time.

Complex *rac*-**1** inhibited the growth of *S. aureus* with MIC and MBC values of 3.60 and 7.19 μM. As the MBC/MIC ratio of complex **1** is approximately 2, complex **1** is considered to be bactericidal. Moreover, complex **1** also exhibited cytotoxicity against a number of cancer cells, with particular potency against ovarian, cervical and melanoma cells but not hepatocarcinoma cells, suggesting that its cytotoxic activity was cell type-dependent. To our knowledge, this cyclometallated iridium(III) complex is the first example of a substitutionally-inert, Group 9 organometallic compound utilized as a direct inhibitor of *S. aureus*.

We anticipate that cyclometallated iridium(III) complex **1** may serve as a useful scaffold for the further development of highly potent inhibitors of *S. aureus* as potential anti-bacterial agents and anti-cancer agents.

## Additional Information

**How to cite this article**: Lu, L. *et al.* Identification of an iridium(III) complex with anti-bacterial and anti-cancer activity. *Sci. Rep.* **5**, 14544; doi: 10.1038/srep14544 (2015).

## Supplementary Material

Supplementary Information

## Figures and Tables

**Figure 1:**
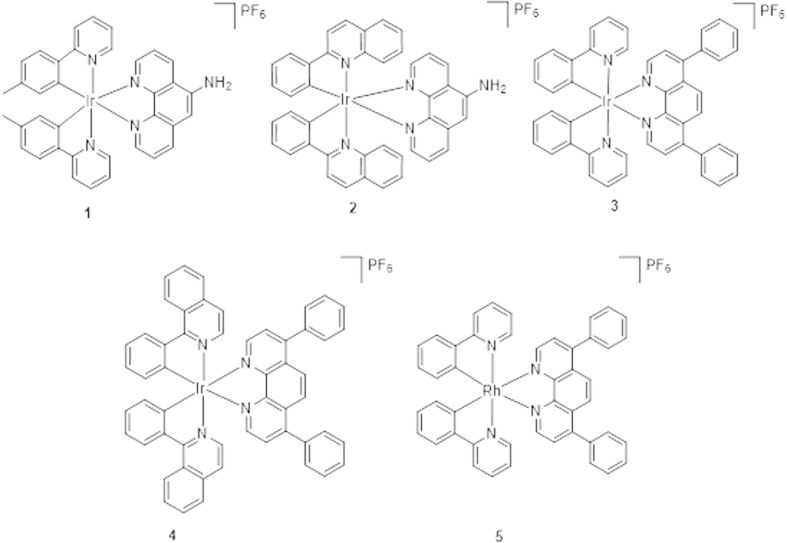
Chemical structures of complexes **1**–**5**.

**Table 1:** Photophysical properties of complexes **1**–**5**.

Complex	λ_em_/nm	UV/vis absorption λ_abs_/nm (*ε*/dm^3^mol^–1^cm^–1^)
**1**	555	270 (2.15 × 10^5^), 381 (2.47 × 10^4^)^[36]^
**2**	590	274 (7.98 × 10^4^), 354 (1.13 × 10^4^)
**3**	587	270 (6.1 × 10^4^), 337 (1.99 × 10^4^)^[37]^
**4**	593	279 (2.0 × 10^5^), 382 (1.56 × 10^4^), 439 (6 × 10^3^)^[38]^
**5**	No emission	284 (1.21 × 10^5^), 366 (2.26 × 10^4^)

## References

[b1] Frezza M. *et al.* Novel metals and metal complexes as platforms for cancer therapy. Curr. Pharm. Des. 16, 1813–1825 (2010). 20337575 10.2174/138161210791209009 PMC3759287

[b2] Howerton B. S., Heidary D. K. & Glazer E. C. Strained ruthenium complexes are potent light-activated anticancer agents. J. Am. Chem. Soc. 134, 8324–8327 (2012). 22553960 10.1021/ja3009677

[b3] Leung C.-H., Zhong H.-J., Chan D. S.-H. & Ma D.-L. Bioactive iridium and rhodium complexes as therapeutic agents. Coord. Chem. Rev. 257, 1764–1776 (2013).

[b4] Knoll J. D. & Turro C. Control and utilization of ruthenium and rhodium metal complex excited states for photoactivated cancer therapy. Coord. Chem. Rev. 282–283, 110–126 (2015). 10.1016/j.ccr.2014.05.018 PMC4343038 25729089

[b5] Ma D.-L., He H.-Z., Leung K.-H., Chan D. S.-H. & Leung C.-H. Bioactive luminescent transition-metal complexes for biomedical applications. Angew. Chem. Int. Ed. 52, 7666–7682 (2013). 10.1002/anie.201208414 23765907

[b6] Heffern M. C., Yamamoto N., Holbrook R. J., Eckermann A. L. & Meade T. J. Cobalt derivatives as promising therapeutic agents. Curr. Opin. Chem. Biol. 17, 189–196 (2013). 23270779 10.1016/j.cbpa.2012.11.019 PMC3622775

[b7] Liu L.-J. *et al.* An iridium(III) complex inhibits JMJD2 activities and acts as a potential epigenetic modulator. J. Med. Chem. 58, 6697–6703 (2015). 26225543 10.1021/acs.jmedchem.5b00375

[b8] Wong C.-Y. *et al.* Dual Inhibition and Monitoring of Beta-Amyloid Fibrillation by a Luminescent Iridium(III) Complex. Curr. Alzheimer Res. 12, 439–444 (2015). 25938873 10.2174/1567205012666150504144558

[b9] Leung C.-H., Lin S., Zhong H.-J. & Ma D.-L. Metal complexes as potential modulators of inflammatory and autoimmune responses. Chem. Sci. 6, 871–884 (2015). 10.1039/c4sc03094j PMC5472922 28660015

[b10] Leung C.-H. *et al.* A metal-based tumour necrosis factor-alpha converting enzyme inhibitor. Chem. Commun. 51, 3973–3976 (2015). 10.1039/c4cc09251a 25610924

[b11] Ma D.-L. *et al.* Antagonizing STAT3 Dimerization with a Rhodium(III) Complex. Angew. Chem. Int. Ed. 53, 9178–9182 (2014). 10.1002/anie.201404686 24889897

[b12] Leung C.-H. *et al.* Inhibition of Janus kinase 2 by cyclometalated rhodium complexes. Medchem. comm 3, 696–698 (2012).

[b13] Zhong H.-J., Yang H., Chan DS.-H., Leung C.-H., Wang H.-M. & Ma D.-L. A metal-based inhibitor of NEDD8-activating enzyme. Plos One 7, (2012). 10.1371/journal.pone.0049574 PMC3501507 23185368

[b14] Leung C.-H. *et al.* A metal-based inhibitor of tumor necrosis factor-alpha. Angew. Chem. Int. Ed. 51, 9010–9014 (2012). 10.1002/anie.201202937 22807261

[b15] Liu J., Sun RW.-Y., Leung C.-H., Lok C.-N. & Che C.-M. Inhibition of TNF-alpha stimulated nuclear factor-kappa B (NF-kappa B) activation by cyclometalated platinum(II) complexes. Chem. Commun. 48, 230–232 (2012). 10.1039/c1cc15317j 22085845

[b16] Wang P. *et al.* Specific blocking of CREB/DNA binding by cyclometalated platinum(II) complexes. Angew. Chem. Int. Ed. 50, 2554–2558 (2011). 10.1002/anie.201006887 21370336

[b17] Spellberg B. *et al.* Combating antimicrobial resistance: policy recommendations to save lives. Clin. Infect. Dis. 52 **Suppl 5**, S397–428 (2011). 21474585 10.1093/cid/cir153 PMC3738230

[b18] Chetana P. R. *et al.* Novel ligand 1-benzyl-3-(4-ethyl-pyridin-2-Yl)-thiourea and Cu(I) complexes: DNA interaction, antibacterial and thermal studies. IJPSDR 21, 355–363 (2013).

[b19] Bormio N. J. H., De Paiva R. E. F., Cuin A., Lustri W. R. & Corbi P. P. Silver complexes with sulfathiazole and sulfamethoxazole: Synthesis, spectroscopic characterization, crystal structure and antibacterial assays. Polyhedron 85, 437–444 (2015).

[b20] Sabounchei S. J. *et al.* New chlorine bridged binuclear silver(I) complexes of bidentate phosphorus ylides: Synthesis, spectroscopy, theoretical and anti-bacterial studies. Polyhedron 85, 652–664 (2015).

[b21] Gholivand K., Molaei F., Oroujzadeh N., Mobasseri R. & Naderi-Manesh H. Two novel Ag(I) complexes of N-nicotinyl phosphoric triamide derivatives: Synthesis, X-ray crystal structure and *in vitro* antibacterial and cytotoxicity studies. Inorganica Chim. Acta 423, 107–116 (2014).

[b22] Yata P. K. *et al.* Study of DNA light switch Ru(II) complexes: synthesis, characterization, photocleavage and antimicrobial activity. J. Fluoresc. 22, 835–847 (2012). 22194001 10.1007/s10895-011-1018-9

[b23] Pathak A., Blair V. L., Ferrero R. L., Mehring M. & Andrews P. C. Bismuth(III) benzohydroxamates: powerful anti-bacterial activity against Helicobacter pylori and hydrolysis to a unique Bi_34_ oxido-cluster [Bi_34_O_22_(BHA)_22_(H-BHA)_14_(DMSO)_6_]. Chem. Commun. 50, 15232–15234 (2014). 10.1039/c4cc07329k 25341969

[b24] Reddy G. N. R., Kondaiah S., Setty K. N., Rao R. M. & Ramulu J. S. Synthesis, structural characterization and anti-bacterial activity of some novel schiff base ligand and their vanadium (IV) metal complexes. OJC 28, 1673–1683 (2012).

[b25] Jain S., Jain N. K. & Pitre K. S. Bio-inorganic studies on the Fe(II) sparfloxacin complex. Met-Based Drugs 9, 1–8 (2002). 18475420 10.1155/MBD.2002.1 PMC2365298

[b26] Kizilcikli I. *et al.* Antimicrobial activity of a series of thiosemicarbazones and their Zn(II) and Pd(II) complexes. Folia Microbiol. (Praha) 52, 15–25 (2007). 17571790 10.1007/BF02932132

[b27] Nair M. S. & Joseyphus R. S. Synthesis and characterization of Co(II), Ni(II), Cu(II) and Zn(II) complexes of tridentate Schiff base derived from vanillin and DL-alpha-aminobutyric acid. Spectrochim. Acta. A. Mol. Biomol. Spectrosc. 70, 749–753 (2008). 17964848 10.1016/j.saa.2007.09.006

[b28] Kantouch A. & El-Sayed A. A. Polyvinyl pyridine metal complex as permanent antimicrobial finishing for viscose fabric. Int. J. Biol. Macromol. 43, 451–455 (2008). 18835405 10.1016/j.ijbiomac.2008.08.011

[b29] Pandurangan K., Gallagher S., Morgan G. G., Muller-Bunz H. & Paradisi F. Structure and antibacterial activity of the silver(I) complex of 2-aminophenoxazine-3-one. Metallomics 2, 530–534 (2010). 21072337 10.1039/c003515g

[b30] Reddy G. N. R., Kondaiah S., Babu P. & Kumar K. R. Synthesis, characterization, and antibacterial activity of the Schiff Base derived from P-toluic hydrazide and 2-hydroxy-4-methoxy acetophenone (HMAPPTH Ligand) and their Mn(II), Co(II), Ni(II) and Cu(II) complexes. JOAC 2, 415–425 (2013).

[b31] Lemire J. A., Harrison J. J. & Turner R. J. Antimicrobial activity of metals: mechanisms, molecular targets and applications. Nat. Rev. Microbiol. 11, 371–384 (2013). 23669886 10.1038/nrmicro3028

[b32] Pandrala M. *et al.* Chlorido-containing ruthenium(II) and iridium(III) complexes as antimicrobial agents. Dalton Trans. 42, 4686–4694 (2013). 23360972 10.1039/c3dt32775b

[b33] Subhan M. A., Alam K., Rahaman M. S., Rahman M. A. & Awal M. R. Synthesis and characterization of metal complexes containing curcumin (C_21_H_20_O_6_) and study of their anti-microbial activities and DNA binding. J. Sci. Res. 6, 97–109 (2014).

[b34] Prasanthi G. Synthesis, characterization and anti-bacterial evaluation of metal complexes of 2-substituted quinazolin-4(3H)-one oxime derivatives. Int. J. Res. Pharm. Sci. 5, 29–31 (2014).

[b35] Bansod N. H., Chaudhari G. N., Thihale M. S. & Patil S. D. Synthesis and antibacterial activity of new complexes of benzothiazole derivatives. Der. Pharma. Chemica. 5, 144–148 (2014).

[b36] Al-Rubaie A. Z. *et al.* Synthesis, characterization and antibacterial activity of some new ferrocenyl selenazoles and 3,5-diferrocenyl-1,2,4-selenadiazole. J. Organomet. Chem. 774, 43–47 (2014).

[b37] Lemire J. A., Harrison J. J. & Turner R. J. Antimicrobial activity of metals: mechanisms, molecular targets and applications. Nat. Rev. Microbiol. 11, 371–384 (2013). 23669886 10.1038/nrmicro3028

[b38] Sumner E. R. *et al.* Oxidative protein damage causes chromium toxicity in yeast. Microbiology 151, 1939–1948 (2005). 15942001 10.1099/mic.0.27945-0

[b39] Helbig K., Grosse C. & Nies D. H. Cadmium toxicity in glutathione mutants of Escherichia coli. J. Bacteriol. 190, 5439–5454 (2008). 18539742 10.1128/JB.00272-08 PMC2493267

[b40] Banin E. *et al.* The potential of desferrioxamine-gallium as an anti-Pseudomonas therapeutic agent. Proc. Natl. Acad. Sci. USA 105, 16761–16766 (2008). 18931304 10.1073/pnas.0808608105 PMC2575493

[b41] Rogers H. J., Woods V. E. & Synge C. Antibacterial effect of the scandium and indium complexes of enterochelin on Escherichia coli. J. Gen. Microbiol. 128, 2389–2394 (1982). 6218256 10.1099/00221287-128-10-2389

[b42] Stojiljkovic I., Kumar V. & Srinivasan N. Non-iron metalloporphyrins: potent antibacterial compounds that exploit haem/Hb uptake systems of pathogenic bacteria. Mol. Microbiol. 31, 429–442 (1999). 10027961 10.1046/j.1365-2958.1999.01175.x

[b43] Olakanmi O. *et al.* Gallium disrupts iron uptake by intracellular and extracellular Francisella strains and exhibits therapeutic efficacy in a murine pulmonary infection model. Antimicrob. Agents Chemother. 54, 244–253 (2010). 19917753 10.1128/AAC.00655-09 PMC2798485

[b44] Banin E. *et al.* The potential of desferrioxamine-gallium as an anti-Pseudomonas therapeutic agent. Proc. Natl. Acad. Sci. USA 105, 16761–16766 (2008). 18931304 10.1073/pnas.0808608105 PMC2575493

[b45] Falzone N., Bohm L., Swarts J. C. & Van Rensburg C. E. Radiosensitization of CHO cells by two novel rhodium complexes under oxic and hypoxic conditions. Anticancer Res. 26, 147–152 (2006). 16475691

[b46] Rajput J. *et al.* Synthesis, characterization and cytotoxicity of some palladium(II), platinum(II), rhodium(I) and iridium(I) complexes of ferrocenylpyridine and related ligands. Crystal and molecular structure of trans-dichlorobis(3-ferrocenylpyridine)palladium(II). J. Organomet. Chem. 689, 1553–1568 (2004).

[b47] Simpson P. V., Schmidt C., Ott I., Bruhn H. & Schatzschneider U. Synthesis, Cellular Uptake and Biological Activity Against Pathogenic Microorganisms and Cancer Cells of Rhodium and Iridium N-Heterocyclic Carbene Complexes Bearing Charged Substituents. Eur. J. Inorg. Chem. 2013, 5547–5554 (2013).

[b48] Mukherjee T. *et al.* Synthesis, characterization, interactions with DNA and bovine serum albumin (BSA), and antibacterial activity of cyclometalated iridium(III) complexes containing dithiocarbamate derivatives. J. Coord. Chem. 67, 2643–2660 (2014).

[b49] Liu Z. *et al.* Contrasting Reactivity and Cancer Cell Cytotoxicity of Isoelectronic Organometallic Iridium(III) Complexes. Inorg. Chem. 50, 5777–5783 (2011). 21618978 10.1021/ic200607j

[b50] Gothe Y., Marzo T., Messori L. & Metzler-Nolte N. Cytotoxic activity and protein binding through an unusual oxidative mechanism by an iridium(i)-NHC complex. Chem. Commun. 51, 3151–3153 (2015). 10.1039/c4cc10014j 25605442

[b51] Lo K. K. *et al.* New luminescent cyclometalated iridium(III) diimine complexes as biological labeling reagents. Inorg. Chem. 42, 6886–6897 (2003). 14552640 10.1021/ic0346984

[b52] Goldsmith J. I., Hudson W. R., Lowry M. S., Anderson T. H. & Bernhard S. Discovery and high-throughput screening of heteroleptic iridium complexes for photoinduced hydrogen production. J. Am. Chem. Soc. 127, 7502–7510 (2005). 15898800 10.1021/ja0427101

[b53] Lu L. *et al.* Detection of nicking endonuclease activity using a G-quadruplex-selective luminescent switch-on probe. Chem. Sci. 5, 4561–4568 (2014).

[b54] Pankey G. A. & Sabath L. D. Clinical relevance of bacteriostatic versus bactericidal mechanisms of action in the treatment of Gram-positive bacterial infections. Clin. Infect. Dis. 38, 864–870 (2004). 14999632 10.1086/381972

[b55] Marmion C. J. *et al.* Combating Cancer with Precious Metals - A Multi-Functional Approach Towards Developing Novel Anti-Cancer Agents. J. Biol. Inorg. Chem. 19, S463–S463 (2014).

[b56] Cheng M., Huang J. X., Ramu S., Butler M. S. & Cooper M. A. Ramoplanin at bactericidal concentrations induces bacterial membrane depolarization in Staphylococcus aureus. Antimicrob. Agents Chemother. 58, 6819–6827 (2014). 25182650 10.1128/AAC.00061-14 PMC4249368

[b57] Dorrani M. *et al.* TXA497 as a topical antibacterial agent: Comparative antistaphylococcal, skin deposition, and skin permeation studies with mupirocin. Int. J. Pharm. 476, 199–204 (2014). 25263100 10.1016/j.ijpharm.2014.09.033

[b58] Louie A. *et al.* Pharmacodynamic evaluation of the activities of six parenteral vancomycin products available in the United States. Antimicrob. Agents Chemother. 59, 622–632 (2015). 25385113 10.1128/AAC.03710-14 PMC4291350

[b59] Oh D. *et al.* Antibacterial activities of amphiphilic cyclic cell-penetrating peptides against multidrug-resistant pathogens. Mol. Pharm. 11, 3528–3536 (2014). 25157458 10.1021/mp5003027 PMC4186684

[b60] Li N. *et al.* PA-1, a novel synthesized pyrrolizidine alkaloid, inhibits the growth of Escherichia coli and Staphylococcus aureus by damaging the cell membrane. J. Antibiot. 67, 689–696 (2014). 24894184 10.1038/ja.2014.49

[b61] Novohradsky V. *et al.* Mechanism of cellular accumulation of an iridium(III) pentamethylcyclopentadienyl anticancer complex containing a C,N-chelating ligand. Metallomics 6, 682–690 (2014). 24448555 10.1039/c3mt00341h

