# Supplementary material for: Identification of an iridium(III) complex with anti-bacterial and anti-cancer activity
Source: Sci Rep. 2015 Sep 29;5:14544. doi: 10.1038/srep14544 (PMC4586517; doi:10.1038/srep14544)
Supplement: Supplementary Information [file srep14544-s1.doc]

**Identification of an iridium(III) complex with anti-bacterial and anti-cancer activity**

Lihua Lu^1,‡^, Li-Juan Liu^2,‡^, Wei-chieh Chao^3,‡^, Hai-Jing Zhong^2^, Modi Wang^1^, Xiu-Ping Chen^2^, Jin-Jian Lu^2^, Ruei-nian Li^3,*^, Dik-Lung Ma^1,*^ & Chung-Hang Leung^2,*^

^1^ Department of Chemistry, Hong Kong Baptist University, Kowloon Tong, Hong Kong, China.

^2^ State Key Laboratory of Quality Research in Chinese Medicine, Institute of Chinese Medical Sciences, University of Macau, Macao, China

^3^ Department of Biomedical Science and Environmental Biology, College of Life Science, Kaohsiung Medical University, Kaohsiung, Taiwan

* Correspondence should be addressed to ([runili@kmu.edu.tw](mailto:runili@kmu.edu.tw); [edmondma@hkbu.edu.hk](mailto:edmondma@hkbu.edu.hk); [duncanleung@umac.mo](mailto:duncanleung@umac.mo))


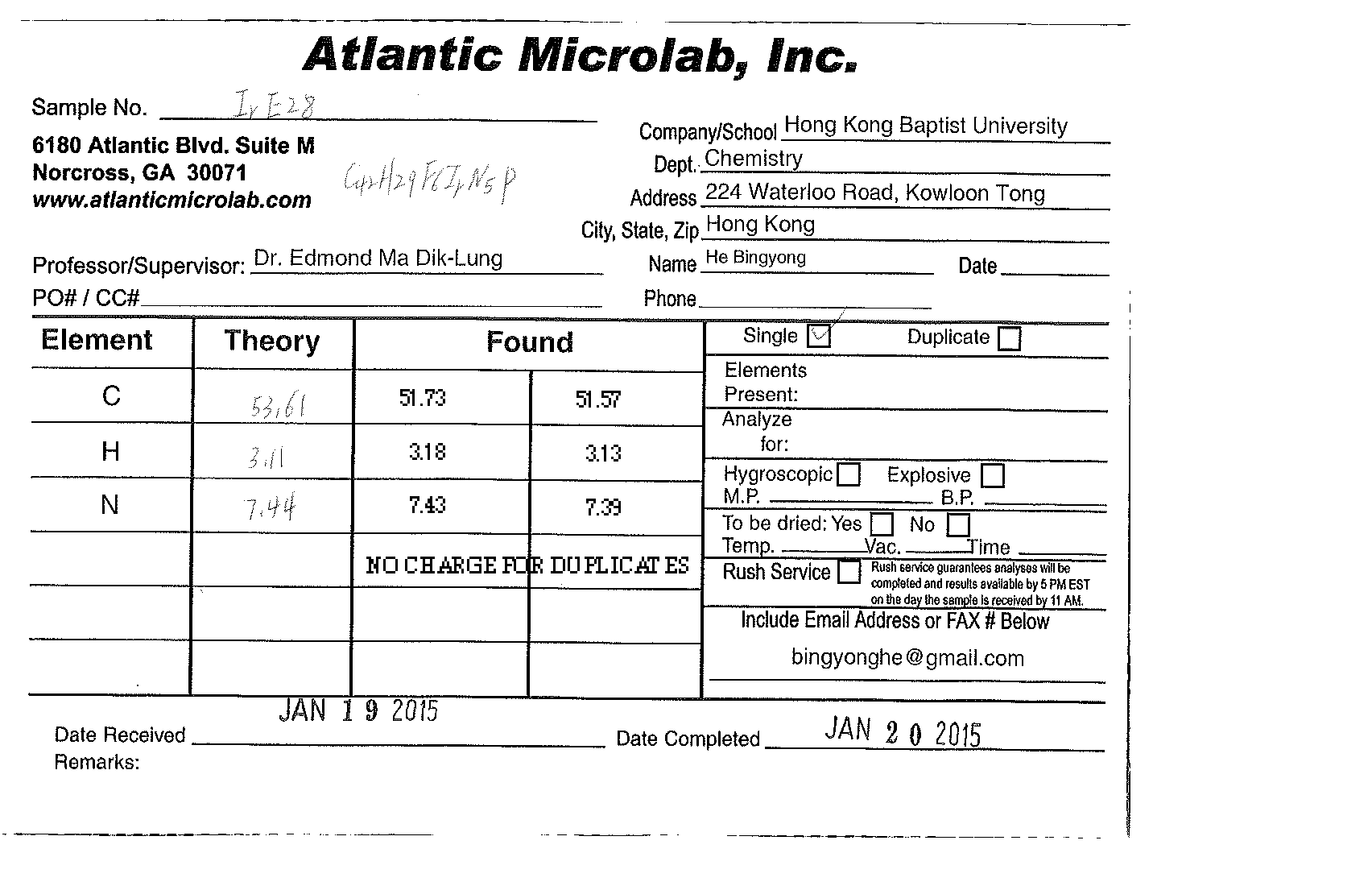


**Figure S1.** Elemental analysis result of complex **2**.


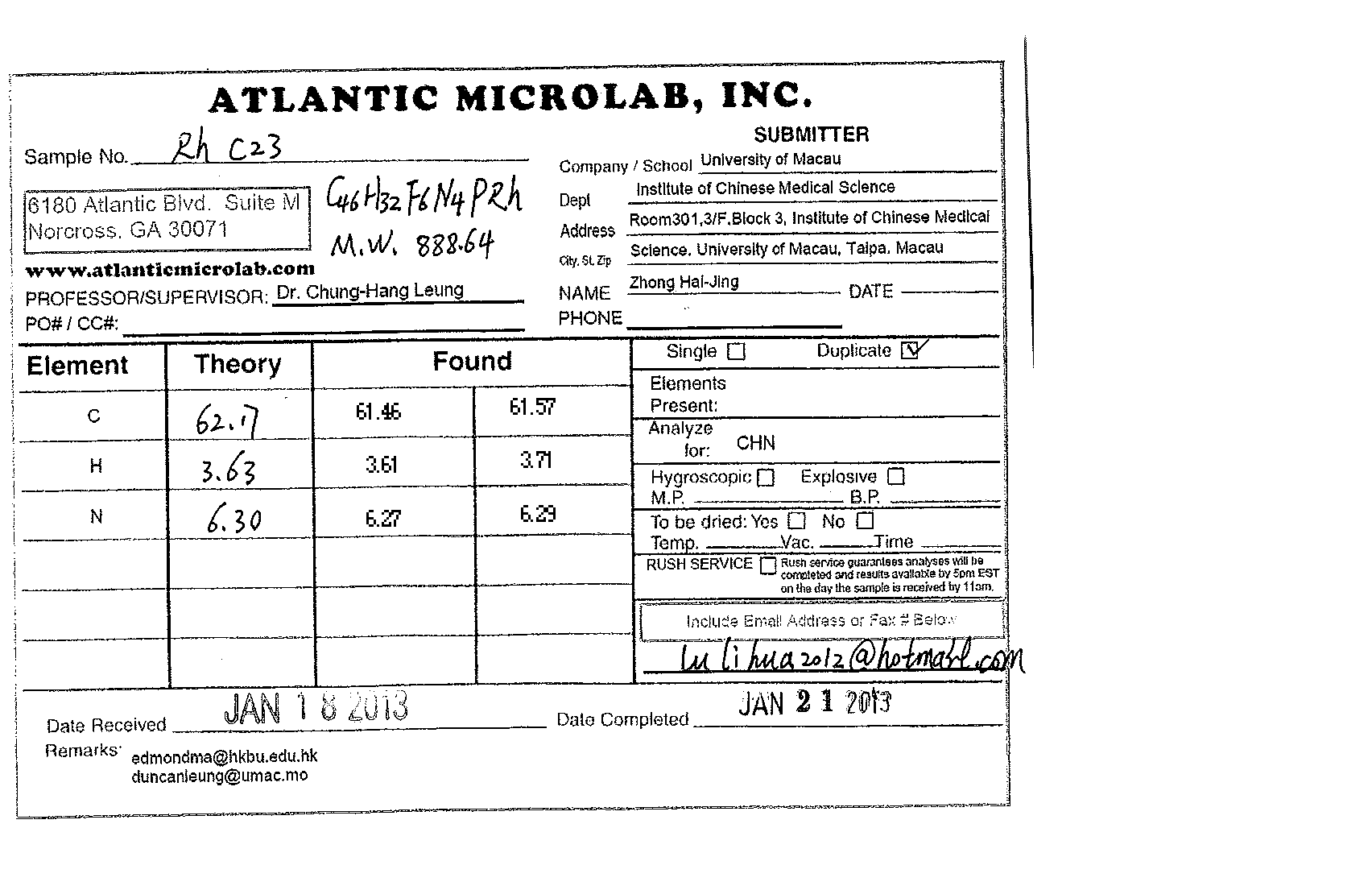


**Figure S2.** Elemental analysis result of complex **5**.

**Table S2.** MIC and MBC values of complex **1** against *S*. *aureus*.

| Conc/µM | 57.53 | 28.76 | 14.38 | 7.19 | 3.60 | 0 |
| --- | --- | --- | --- | --- | --- | --- |
| Test 1 (CFU) | 0 | 0 | 0 | 0 | 100 | 10^9^ |
| Test 2  (CFU) | 0 | 0 | 0 | 0 | 1438 | 1.14x10^11^ |
| Test 3  (CFU) | ND | 0 | 0 | 1 | 438 | 10^9^ |

Conc: Concentration

CFU: Colony forming units

ND: Not determined
